# Porous Polymeric Nanofilms for Recreating the Basement Membrane in an Endothelial Barrier-on-Chip

**DOI:** 10.1021/acsami.3c16134

**Published:** 2024-02-28

**Authors:** Elena Mancinelli, Nanami Zushi, Megumi Takuma, Chalmers Chi Cheng Chau, George Parpas, Toshinori Fujie, Virginia Pensabene

**Affiliations:** † School of Electronic and Electrical Engineering and Pollard Institute, University of Leeds, Leeds LS2 9JT, United Kingdom; ‡ Bragg Centre for Materials Research, University of Leeds, Leeds LS2 9JT, United Kingdom; § School of Life Science and Technology, Tokyo Institute of Technology, B-50, Nagatsuta-cho, Midori-ku, Yokohama 226-8501, Japan; ∥ School of Molecular and Cellular Biology and Astbury Centre for Structural Molecular Biology, University of Leeds, Leeds LS2 9JT, United Kingdom; ⊥ Leeds Institute of Biomedical and Clinical Sciences, University of Leeds, Leeds LS2 9JT, United Kingdom; # Living Systems Materialogy (LiSM) Research Group, International Research Frontiers Initiative (IRFI), Tokyo Institute of Technology, R3-23, 4259 Nagatsuta-cho, Midori-ku, Yokohama 226-8503, Japan; ∇ Faculty of Medicine and Health, Leeds Institute of Medical Research at St James’s University Hospital, University of Leeds, Leeds LS2 9JT, United Kingdom

**Keywords:** organ-on-a-chip, semipermeable inserts, endothelial barrier, basement barrier, porous polymeric nanofilms

## Abstract

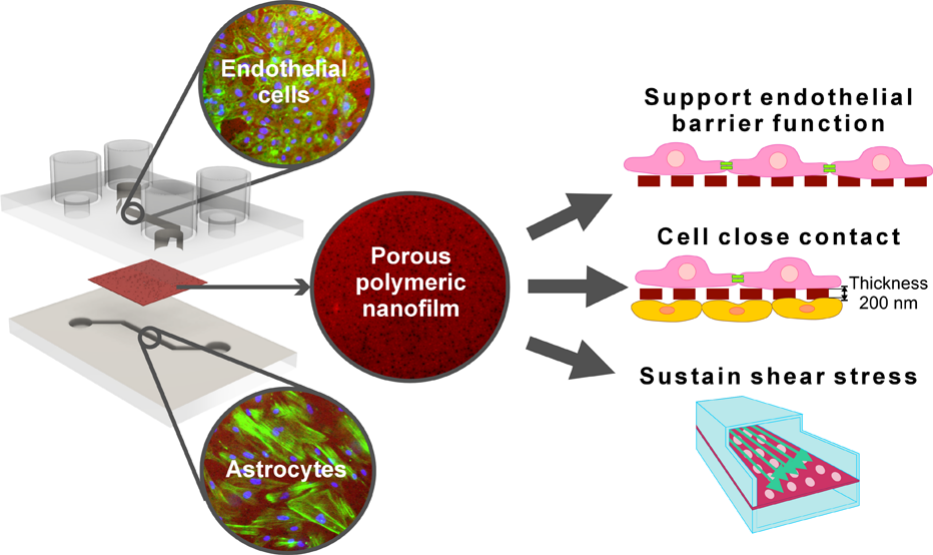

Organs-on-chips (OoCs) support an organotypic human cell culture *in vitro*. Precise representation of basement membranes (BMs) is critical for mimicking physiological functions of tissue interfaces. Artificial membranes in polyester (PES) and polycarbonate (PC) commonly used in *in vitro* models and OoCs do not replicate the characteristics of the natural BMs, such as submicrometric thickness, selective permeability, and elasticity. This study introduces porous poly(d,l-lactic acid) (PDLLA) nanofilms for replicating BMs in *in vitro* models and demonstrates their integration into microfluidic chips. Using roll-to-roll gravure coating and polymer phase separation, we fabricated transparent ∼200 nm thick PDLLA films. These nanofilms are 60 times thinner and 27 times more elastic than PES membranes and show uniformly distributed pores of controlled diameter (0.4 to 1.6 μm), which favor cell compartmentalization and exchange of large water-soluble molecules. Human umbilical vein endothelial cells (HUVECs) on PDLLA nanofilms stretched across microchannels exhibited 97% viability, enhanced adhesion, and a higher proliferation rate compared to their performance on PES membranes and glass substrates. After 5 days of culture, HUVECs formed a functional barrier on suspended PDLLA nanofilms, confirmed by a more than 10-fold increase in transendothelial electrical resistance and blocked 150 kDa dextran diffusion. When integrated between two microfluidic channels and exposed to physiological shear stress, despite their ultrathin thickness, PDLLA nanofilms upheld their integrity and efficiently maintained separation of the channels. The successful formation of an adherent endothelium and the coculture of HUVECs and human astrocytes on either side of the suspended nanofilm validate it as an artificial BM for OoCs. Its submicrometric thickness guarantees intimate contact, a key feature to mimic the blood–brain barrier and to study paracrine signaling between the two cell types. In summary, porous PDLLA nanofilms hold the potential for improving the accuracy and physiological relevance of the OoC as *in vitro* models and drug discovery tools.

## 1 Introduction

Organs-on-chips (OoCs) are sophisticated microfluidic models that replicate the essential functional units of tissues and organs.^1^ In its simplest configuration, an OoC consists of a perfused microfluidic chamber housing a single cell type. However, more complex scenarios require the use of multiple microchambers to recreate the physiological interactions and dynamics between tissues.^2^

Key constituents of tissue interfaces are extracellular matrix (ECM) components and a wide range of cell types. Among these, epithelial and endothelial cells fulfill the vital role of barrier, effectively protecting sensitive areas of our body including brain,^3^ retina,^4^ kidneys,^5^ intestine,^6^ and lungs.^7,8^ The growth of endothelial and epithelial cells is facilitated by the basement membrane (BM), a thin (∼100 nm for most BMs in the body^9,10^) but dense and elastic (reported Young’s modulus from kPa^10^ to single-digit MPa range^9^) layer of ECM proteins. The BM lines the basolateral side of the epithelium and the endothelium, providing essential support for cellular separation and enabling communication.^9^

Hence, investigating cellular barriers at the tissue interfaces necessitates the development of *in vitro* coculture systems that incorporate artificial representations of BMs.

In the past decades, microfluidic-based endothelial barrier models have emerged alongside static Transwell-based coculture systems. Microfluidic chips allow for the accurate recapitulation *in vitro* of the mechanical stimuli experienced by the endothelium along vessel walls, including continuous shear stress generated by blood flow.^11−13^ This stress plays a pivotal role in enhancing the integrity of the endothelial barrier.^14^ Moderate levels of shear stress (up to 15 dyn/cm^2^) keep endothelial cells in a nonproliferative and noninflammatory state.^14,15^ Furthermore, under laminar and unidirectional flow, endothelial cells align themselves in the direction of flow, reinforcing their cohesion and the structural integrity of the barrier.^15^

An immediate model of BM structure and functions is based on a stack of 2 or more microfluidic compartments communicating through semipermeable inserts.^8,16,17^ Selecting or synthesizing suitable inserts for this purpose is a complex, multiparametric task. First of all, the permeable substrate needs to faithfully replicate both the biochemical and biophysical BM properties^18−20^ to support cell adhesion, proliferation, and differentiation.^21,22^ Practical manufacturing considerations, such as ease of handling, scalability, compatibility with microfluidic systems, and ability to consistently sustain appropriate shear stress levels, are also important. As a result, flat polymeric porous membranes have been consistently preferred over other configurations that bear a closer resemblance to physiological conditions. For example, hydrogel-based three-dimensional cultures^23^ hinder high-resolution imaging,^24^ electrospun nanofiber membranes are ill-suited for load-bearing applications,^25^ and vitrified ECM protein membranes^26^ exhibit low synthesis reproducibility.^17^

A significant volume of data is available for track-etched membranes^27^ made of polyester (PES) and polycarbonate (PC). These inserts constitute an essential component of Transwell assays and are available as standalone for integration into OoCs.^28−30^ However, they do not accurately replicate BM properties in terms of thickness and porosity. The thickness of track-etched membranes (>10 μm) is considerably greater than vascular BMs.^9^ This hinders cell paracrine signaling and reduces membrane permeability. To prevent the formation of undesired large pores resulting from merged ion tracks, track-etched membrane porosity is also intentionally kept low.^27^ Pores larger than 3 μm can cause compartmentalization failure and cell extravasation.^31^ Limited optical transparency or autofluorescence^32,33^ also hinders compatibility of PES and PC with bright field microscopy.

Biocompatible poly(dimethylsiloxane) (PDMS) membranes address transparency issues and offer adjustable stiffness. As a result, they are commonly used in OoCs for all-PDMS device fabrication.^34,35^ However, PDMS membranes, with a minimum thickness of 1 μm,^36^ remain difficult to manufacture at large scale and unsuitable for studying endothelial barriers such as the blood–brain barrier (BBB), where direct contact occurs between astrocyte endfeet and brain capillary endothelium.^37,38^

Super-thin silicon-based molecular filters^39,40^ with thicknesses ranging from 15 to 400 nm are intrinsically transparent and thus suited for cell coculture experiments.^18,41−43^ Their mechanical hardness and intrinsic fragility pose limitations for integration into PDMS-based devices and require the use of supporting frame materials for handling.^44^

Biodegradable polymeric nanofilms represent a promising category of nanomaterials for various biomedical applications^45^ including the replication of BMs in OoCs.^46^ Their nanometer-scale thickness closely resembles physiological BMs, their transparency allows for compatibility with bright field microscopy, and their biocompatibility has been already demonstrated with different cell types.^45,47−49^ With thicknesses ranging from tens to hundreds of nanometers, they exhibit an exceptionally high lateral dimension-to-thickness ratio, approaching approximately 10.^6,45^ Consequently, they possess properties similar to those of 2D soft materials, such as noncovalent adhesion to diverse substrates,^50^ adjustable flexibility, mechanical strength,^51^ and unique conductive properties.^52^ Recent advancements in thin coating techniques, like spin coating or roll-to-roll (R2R) gravure coating, combined with polymer phase separation^53^ and vapor-induced phase separation,^54,55^ have enabled the synthesis of porous polymeric nanofilms. Unlike the standard spin-coating protocol, the R2R technique enables the large-scale synthesis of porous polymeric nanofilms, a crucial parameter for expediting the overall manufacturing process. Furthermore, coupling with the polymer phase separation process eliminates the need for additional steps required to control vapor exposure in the process of vapor-induced phase separation. In our previous study, we examined the compatibility of porous poly(d,l-lactic acid) (PDLLA) nanofilms, fabricated combining R2R gravure coating and polymer phase separation, as substrates for endothelial cell culture, and we have provided a procedure for integrating these into a dual-chamber microfluidic system.^56^ In this work, we assessed their off-chip characteristics as an artificial BM replica, including thickness, porosity, permeability, and Young’s modulus. Using human umbilical endothelial cells (HUVECs) as a model of endothelial cells, we first showed cell proliferation and barrier formation into an open microfluidic device and Transwell inserts that integrate PDLLA nanofilms. We benchmarked these nanofilms against a commercially available PES track-etched membrane and then designed a PDMS endothelial barrier-on-chip where the porous nanofilm serves as a submicrometer-thick permeable substrate separating stacked microfluidic channels. After confirming the establishment of a coherent monolayer of endothelial cells on one side of the nanofilm, we then introduced human astrocytes on the other side of the nanofilm into the other microfluidic compartment. The coculture of endothelial cells and astrocytes, separated only by the ultrathin “artificial” basal membrane, recreates the physiological proximity between the two cell types in the blood–brain barrier.

## 2 Materials and Methods

### 2.1 Preparation and Characterization of PDLLA Porous Nanofilms

**Figure 1:**
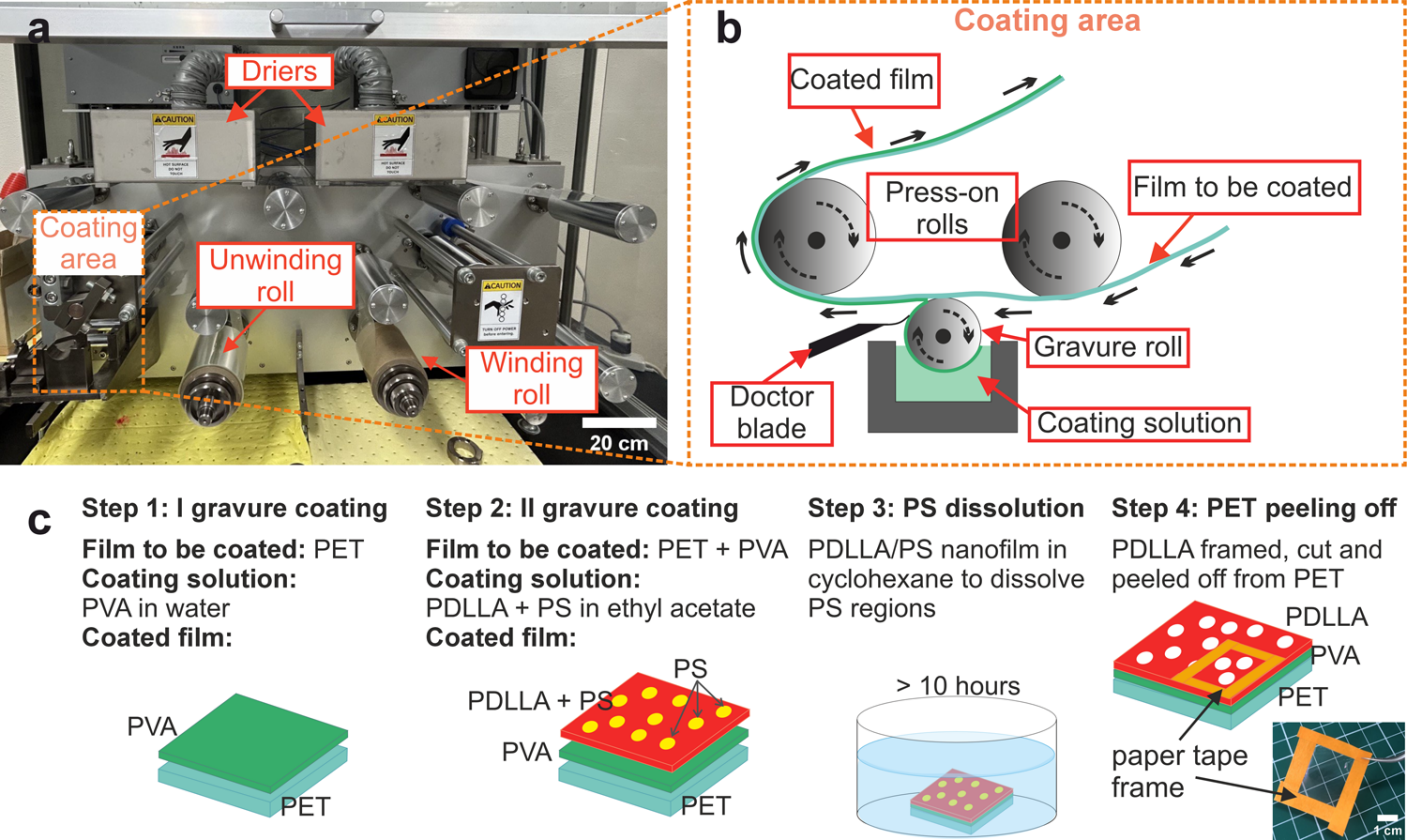
Porous PDLLA nanofilm fabrication by roll-to-roll gravure coating and polymer phase separation: (a) lab equipment for roll-to-roll gravure coating mounting a roll of PET film to be coated (scale bar: 20 cm); (b) schematic overview of roll-to-roll coating procedure; (c) schematic outlining the fabrication protocol for the porous PDLLA nanofilm, which includes roll-to-roll coating (two steps), immersion in cyclohexene for selective PS dissolution, and peeling off the PET support from the PDLLA–PVA sheet. PVA can then be dissolved by immersing the remaining PDLLA–PVA sheet in water.

The fabrication protocol for ultrathin porous films made of PDLLA was detailed by S. Suzuki et al.^53^ In the present study, we used a 40 mg/mL solution of PDLLA (Mw = 300,000–600,000, Polyscience, Inc., Warrington, PA, U.S.A.) and polystyrene (PS, MW = 280,000, Sigma-Aldrich Co., LLC, St. Louis, MO, U.S.A.). PDLLA and PS are equally concentrated in ethyl acetate (Kanto Chemical, Co., Inc., Japan) and serve as precursor polymeric blends for the nanofilms. For ease of handling and transport, nanofilms are attached to a thicker supportive layer of poly(ethylene terephthalate) (PET, Lumirror 25T60, Panac Co., Ltd., Tokyo, Japan) by means of a sacrificial layer of poly(vinyl alcohol) (PVA, Mw = 13,000–23,000, Kanto Chemical, Co., Inc., Japan). The three-layered polymeric sheet (PET–PVA–PDLLA) is assembled by two consecutive gravure coating steps (Micro Gravuret coater ML-120, Yasui Seiki Co., Ltd., Kanagawa, Japan) performed at a line speed of the film of 1.3 m/min and a gravure rotation speed of 30 rpm (Figure 1a–b). Thus, the PET substrate is first coated with PVA, and after a 5 min curing step at 80 °C, the PET–PVA substrate is coated with the PDLLA–PS mixture. The resulting sheet is heated at 80 °C for 5 min. Drying steps are performed by setting the build-in dryers (Figure 1a) at the desired temperature. The sheet is then immersed and sonicated overnight (>10 h) in cyclohexane. Cyclohexane selectively dissolves PS opening pores within the PDLLA nanofilm (Figure 1c). By adding Nile Red (Tokyo Chemical Industry Co., Ltd., Japan) stain in the initial polymer blend (10^–4^ mg/mL in ethyl acetate), the resulting nanofilm becomes fluorescent in red. PET separation from the sheet is performed by peeling off a frame of 4 pieces of overlapping paper tape framing an area of PDLLA (Figure 1c). Alternatively, a free-standing PDLLA nanofilm is obtained by immerging the PET–PVA–PDLLA sheet in deionized water to dissolve the PVA layer.

### 2.2 Height Profile Scanning, AFM Imaging and Scan Analysis

Once floating in water, the PDLLA nanofilm was collected with a glass coverslip providing substrates for height profile scanning and AFM imaging. Polyester (PES) membrane (ipPORE, Belgium, pore size, 1 μm; pore density, 2 × 10^6^/cm^2^; thickness, 11 μm) samples were fixed on equivalent substrates by means of tape frame, ensuring optimal stretching of the membrane. Thickness was evaluated by a DektakXT stylus profilometer (Bruker, MA, U.S.A.). PDLLA nanofilms and PES membranes were imaged using a Bruker Dimension Fastscan (Bruker, MA, U.S.A.) with SCOUT 350 HAR silicon AFM probe (NuNano, Bristol, U.K.) in tapping mode in air with driving amplitude at 17 mV and scan rate at 2 Hz. Images were acquired at a high resolution of 1024 × 1024 samples or higher via NanoScope 9.1 and analyzed with NanoScope Analysis 1.9 software.

### 2.3 Tensile Test

Tensile testing for porous PDLLA nanofilms and PES membranes was performed by a universal testing machine (Shimadzu, Japan). Young’s modulus of the two materials was calculated as the slope of the first linear elastic region of the stress (σ)–strain (ε) curve, defined as
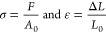
where *F* is equal to the pulling force applied by the machine, *A*_0_ is the original cross-sectional area of the substrate under tension (width × thickness), Δ*L* is the extension stroke detected by the machine, and *L*_0_ is the initial axial length of the substrate under tension.

### 2.4 Contact Angle Measurements

Surface wettability and hydrophilicity of the films were evaluated by static water contact angle measurements using the sessile drop method (OCA 25, Data Physics Corporation, CA, U.S.A.). A 2 μL water drop was dispensed on the nanofilms adhered to glass slides. The angle was evaluated from the recorded frames with the OCA 25 software.

### 2.5 Transwell Insert Assembly and Off-Chip Endothelial Barrier Assessment

Endothelial barrier assessment was performed by mounting suspended PDLLA nanofilms and PES membranes on Transwell inserts (Corning, NY, U.S.A.). After obtaining a free-standing PDLLA nanofilm in deionized water, it was scooped using the membrane-free Transwell insert. Inserts integrating porous PDLLA nanofilm were left at room temperature until fully dried and then securely attached to the inset walls by precisely casted and cured PDMS (schematic of the assembling protocol in Supporting Information Figure S1 and Figure 3a showing Transwell inserts integrating a PDLLA nanofilm). PES membranes were cut to fit the Transwell inserts and attached using liquid PDMS.

Prior to cell seeding, the inserts underwent UV sterilization treatment (254 nm, 25 min) and were coated with bovine fibronectin (FN, Sigma-Aldrich Co., LLC, MO, U.S.A.) diluted in Hank’s balanced salt solution (HBSS, Thermo Fisher Scientific, MA, U.S.A.), at a density of 2.5 μg/cm^2^. Finally, all culturing substrates were filled with a complete endothelial cell medium and equilibrated in an incubator at 37 °C and 5% CO_2_ (∼3 h). After trypsinization and centrifugation, cells were suspended in complete medium and plated at a seeding density of 300 cell/mm^2^. Transendothelial electrical resistance (TEER) measurements were collected by means of an EVOM2 epithelial voltohmmeter integrating standard STX2 electrodes (World Precision Instruments, FL, U.S.A.). Before measurements were collected, electrodes were cleaned with a 70% ethanol solution and dried under laminar flow. To calculate the TEER values, the following formula was used:
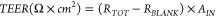
where *R*_*TOT*_ represents the total resistance across the cell monolayer grown on the semipermeable inserts, *R*_*BLANK*_ is the resistance across the porous substrate in medium without cells, and *A*_*IN*_ is the area of the substrate, 1.12 cm^2^ for the 12-well plates used in this study. Cells were cultured for 10 days on the inserts where medium was refreshed 1 h prior to TEER measurements.

Permeability measurements were performed using fluorescein isothiocyanate (FITC)-Dextran (molecular weight: 150 kDa, Sigma-Aldrich Co., LLC, St. Louis, MO). The basolateral side of the Transwell inserts was first filled with 1.5 mL of endothelial cell medium. A working solution of 25 mg/mL FITC-dextran in endothelial cell medium was prepared and used to fill the apical side (300 μL for each insert). The plate was then incubated for 30 min, protected from direct light at room temperature. Permeation was then interrupted by removing the inserts from the wells. The basolateral medium, now containing (FITC)-dextran that crossed the monolayer, was thus collected, and the (FITC)-dextran concentration was measured with a fluorescence (plate) reader (Tecan Group, Ltd., Switzerland) with filters appropriate for 485 and 535 nm excitation and emission, respectively. A standard curve fluorescence of (FITC)-dextran versus concentration was also evaluated, and it is shown in the Supporting Information (Figure S2). Apparent permeability against 150 kDa FITC-dextran (*P*_*app*_) is evaluated as, where *V*_*B*_ is the volume of the bottom well (acceptor), *t* is the time elapsed since the inoculation of the dextran solution into the top well (donator), *A*_*IN*_ is the area of the semipermeable inserts and *C*_*B*_ and *C*_*Ain*_ are, respectively, dextran concentration at the basolateral side and the initial concentration at the apical side of the Transwell. After TEER and permeability measurements at day 5 in culture, we then repetitively shook the well plate at room temperature to disrupt the barrier and measure an additional value of permeability.

### 2.6 Assembly of Microfluidic Devices

Two different designs of double compartment microfluidic devices integrating different semipermeable inserts were assembled: simple open devices (Figure 3c) and fully enclosed double layer microfluidic devices (Figure 5c). Simple open devices to test cell proliferation on suspended porous PDLLA nanofilms and commercially available track-etched PES membranes are composed of a bottom layer consisting of 16 parallel channels (200 μm wide, 20 mm length, and 100 μm deep) and a top open culturing chamber. The bottom channels are obtained by casting and curing (overnight at 65 °C) liquid PDMS (polymer/curing agent, 10:1) on SU8 2075 molds (Kayaku Advanced Materials, MA, U.S.A.). The open culturing chamber is fabricated by punching a 5-mm-thick cured PDMS layer with a 6 mm diameter puncher (WellTech Laboratories, Wellmate Enterprise Co., Ltd., Taiwan). Culturing chambers with a glass bottom were obtained by bonding this layer to a glass slide (Epredia Microscope Slides, Cut, 1 mm, Thermo Fisher Scientific, MA, U.S.A.) by plasma bonding (0.5 mbar, 13.56 MHz, 200 W, 30 s, Diener Electronic GmbH & Co. KG, Germany).

Fully enclosed double layer microfluidic devices recreating endothelial barrier-on-chip consist of a stack of 2 channels (400 μm wide, 10 mm length, and 100 μm high) separated by suspended PDLLA nanofilms and obtained with the same soft lithographic process. Inlet and outlet ports are opened by punching holes of 1.5 mm (Integra Miltex, NJ, U.S.A.). To integrate the PDLLA nanofilms in the devices, the PET–PVA–PDLLA sheet is cut into the desired shape, and the PET layer is lifted off by using 4 overlapping pieces of paper tape enclosing a 1.5 × 1.5 cm^2^ paper tape frame (Figure 1c and Figure S3 in Supporting Information). The remaining PVA–PDLLA sheet and the bottom PDMS compartment undergo oxygen plasma treatment (0.5 mbar, 13.56 MHz, 200 W, 30 s, Diener Electronic GmbH & Co. KG, Germany). The exposed surfaces are gently pressed together and left at 50 °C (>2 h). Only after the bonding between PDLLA and PDMS are the 2 pieces are covered by deionized water to dissolve the PVA sacrificial layer. The PDMS–PDLLA complex is dried at room temperature and bonded with the remaining PDMS compartment, following a second oxygen plasma activation (0.5 mbar, 13.56 MHz, 200 W, 30 s, Diener Electronic GmbH & Co. KG, Germany) of both surfaces. Fully assembled devices are left at 50 °C for at least 2 h and then filled with deionized sterile water (schematic of the assembly process shown in Supporting Information, Figure S3). The PES membrane was integrated between PDMS layers following the procedure described by Aran et al.^57^ by means of a 5% solution of (3-aminopropyl)triethoxysilane (APTES, Sigma-Aldrich, MO, U.S.A.). 500 μL of Pyrex cloning cylinder (Fisher Scientific, PA, U.S.A.) is attached with liquid PDMS to inlet, outlet, and open culturing chambers to provide reservoirs for cell medium. The reservoirs were filled with deionized sterile water before cell seeding. The devices were all kept at 4 °C until used to prevent water evaporation and maintain hydrophilicity acquired by oxygen-plasma treatment.

### 2.7 Flow and Shear Stress Test

Sealing, bonding, and the capability to sustain shear stress were assessed with a flow test. The double channel microfluidic device integrating the PDLLA nanofilm was connected to a syringe pump (KF Technology, Italy) by means of Tygon tubing with internal diameter (ID) 0.020 in. and outer diameter (OD) 0.060 in. (Cole Parmer, IL, U.S.A.) and 24G blunt needle connections (Sai Infusion Technologies, IL, U.S.A.). The spent solution was collected through the outlet tubing. The shear stress solution consisted of polystyrene beads (Sigma-Aldrich, microparticles based on polystyrene, 10 μm) diluted in phosphate-buffered saline solution (PBS). Videos were recorded by means of an inverted phase contrast microscope (VWR, VisiScope IT404, Profcontrol GmbH, Germany) equipped with a camera (GXCAM HiChrome HR4 Lite, GT Vision, U.K.). Average wall shear stress (τ) values were calculated assuming a Newtonian fluid, using the simplified formula for microfluidic perfusion culture in 2D Poiseuille flow systems:
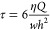
1where η is the dynamic viscosity of water, *Q* is the flow rate, *w* is the channel width, and *h* is the channel height with the assumption of fully developed flows in a channel where *w* is greater than *h*. Nanofilm capability to sustain shear stress was tested at 5, 20, and 80 μL/min.

### 2.8 Cell Culture and Device Seeding

HUVECs (Lonza, Switzerland) and human astrocytes (HAs, ScienCell, CA, U.S.A.) were subcultured in conventional T75 flasks up to passage 10 and maintained in a 37 °C, 5% CO_2_ incubator. HUVECs were cultured in endothelial cell medium supplemented with 1% endothelial cell growth supplement, 5% fetal bovine serum (FBS), and 1% Pen-Strep mixture. HAs were cultured in astrocyte medium (AM) supplemented with 1% astrocyte growth supplement, 5% FBS, and 1% Pen-Strep mixture. Media and supplements were all purchased from ScienCell, CA, U.S.A. Microfluidic devices were UV-sterilized (wavelength: 254 nm) for 25 min and coated prior to seeding. The microfluidic compartment for HUVEC culture was coated with FN (Sigma-Aldrich Co., LLC, MA, U.S.A.) diluted in HBSS (Thermo Fisher Scientific, MA, U.S.A.), at a density of 2.5 μg/cm^2^, while the compartment for the HA culture was coated with laminin (Sigma-Aldrich Co., LLC, MA, U.S.A.) and was diluted in HBSS at a density of 1.5 μg/cm^2^. For exclusive HUVEC culture in open devices, 5 × 10^3^ cells were seeded on each device, while for fully enclosed devices, 1 × 10^6^ cells were suspended into 1 mL of complete endothelial cell medium and loaded inside the top compartment of the device (∼100 μL each device). Cells were allowed to attach for 1 h. In multiple cell type cultures, HUVECs were seeded in the bottom channel using the same procedure, but cell seeding was performed with the device upside down and inlet and outlet ports sealed with cured UV-sterilized PDMS. After attaching, the device was inverted to the upright position, and the medium was added to the inlets. HUVECs were cultured for 5 days, with medium changes occurring twice daily. The medium was replaced by emptying and refilling the inlet reservoir with 500 μL of fresh complete endothelial cell medium. After 5 days, the device was prepared for HA seeding in the top compartment, following the same procedure with an initial cell concentration of 0.50 × 10^6^ cells in 1 mL of complete AM. Simultaneous culture of HAs and HUVECs continued for 5 days before cell fixation and staining.

### 2.9 Device Maintenance under Capillary Flow

In both single and coculture conditions in fully enclosed microfluidic devices, HUVECs experienced capillary flow driven by the tendency of the liquid cell medium to equilibrate between the inlet and outlet reservoirs. The evaluation of shear stress follows eq 1, with the flow rate (*Q*) estimated by the Hagen–Poiseuille equation () where Δ*P* is the difference between the hydrostatic pressures at the inlet and outlet (Δ*P* = *pg*Δ*H*), Δ*H* is the liquid height difference between the inlet and outlet reservoirs, and *R*_*H*_ is the hydrodynamic resistance of the microfluidic channel. *R*_*H*_ can be approximated as  where η is the dynamic viscosity of water, *l* is the length of the channel, *w* is its width, and *h* is its height.^58^ Cells on nanofilm experience the highest shear stress every 12 h when the inlet reservoir is refilled with medium, and Δ*H* is equal to the total height of the reservoir (1 cm). To limit shear stress on HAs, astrocyte medium was replenished every 48 h, and both reservoirs were filled after 5 min to zero out Δ*H* and shear stress.

### 2.10 Staining and Image Acquisition

To complete the proliferation and morphology study of HUVECs, NucBlue Live reagent (Hoechst 33342) (ReadyProbes Cell Viability Imaging Kit (Blue/Red), Molecular Probes, OR, U.S.A.) and actin filament (F-actin) staining (ActinGreen 488 ReadyProbes Reagent, Molecular Probes, OR, U.S.A.) were performed after 3, 5, and 7 days of culture in the open microfluidic devices. Each time, 9 devices were stained: 3 integrating PDLLA nanofilm, 3 integrating PES membrane, and 3 with a glass bottom. Before F-actin staining, cells were washed 3 times in PBS, fixed with 4% paraformaldehyde for 10 min at room temperature, rinsed with PBS, and stained. LIVE/DEAD assay (ReadyProbes Cell Viability Imaging Kit (Blue/Red), Molecular Probes, OR, U.S.A.) was performed on 3 additional culturing chambers on day 7 (2 pictures for each culturing chamber). When cultured within the endothelial–barrier-on-chip, HUVECs were fixed after 5 days of culture and stained to label nuclei and F-actin or nuclei and zonula occludens (ZO)-1 tight junction proteins. For ZO-1 staining, a blocking solution made of 1% bovine serum albumin (Thermo Fisher Scientific, MA, U.S.A.) diluted in 1% PBS was added in the top microfluidic channel and left at room temperature for 30 min; after washing with PBS for 15 min (3 × 5 min/wash), the culture was incubated overnight at 4 °C with primary rabbit ZO-1 antibody (Thermo Fisher Scientific, MA, U.S.A.) diluted 1:100 in blocking solution, followed by 3 washings with PBS (5 min/wash) to remove the unbound antibodies. Alexa Fluor 488 Goat anti-Rabbit IgG (H+L) Cross-Adsorbed Secondary Antibody (Thermo Fisher Scientific, MA, U.S.A.) diluted 1:100 in blocking solution was added to the top microfluidic channel and incubated at room temperature for 1 h under gently agitation. The culture was then washed with PBS 3 times. When seeded with both HUVECs and astrocytes, cells were fixed following a culture period of 10 days for HUVECs and 5 days for astrocytes. Subsequently, both cell types were stained for nuclei and F-actins, while HUVECs were also stained for platelet endothelial cell adhesion molecule (CD31). For CD31 staining, the same protocol as for ZO-1 was applied using a primary mouse monoclonal antibody (antibodies.com, U.K.) diluted 1:100 in blocking solution and secondary Alexa Fluor 647 Cross-Adsorbed Goat Anti-Mouse IgG (H+L) Antibody (Thermo Fisher Scientific, MA, U.S.A.) diluted 1:200 in blocking solution. Bright field and fluorescence images were acquired in phase contrast mode with an inverted microscope (Nikon ECLIPSE Ti2, Nikon Instruments, Inc., NY, U.S.A.) equipped with a Digital CMOS camera (ORCA Flash4.0 V3, Hamamatsu Photonics, Japan) and an LED illumination system (pE-4000 CoolLED, MA, U.S.A.). 3D reconstruction of the devices and ZO-1-stained cells were observed by a confocal laser scanning microscope (Nikon A1R, Nikon Instruments, Inc., NY, U.S.A.).

### 2.11 Image and Data Analysis

Nanofilm thickness was evaluated using MATLAB programming language (The Mathworks, MA, U.S.A.) from height profile traces (one trace for each batch of nanofilms and 3 profile values extracted for each trace, N = 9). Pore diameters (N = 440 across 6 AFM scans for PDLLA and N = 68 across 6 AFM scans for PES), percentage of area covered by pores (porosity) (N = 6 AFM scans), and pore density (N = 6 AFM scans) were evaluated from AFM scans using ImageJ software. Root mean square (Rq) and arithmetic average (Ra) roughness were evaluated on 1 × 1 μm^2^ areas across 3 AFM scans for each different substrate (N = 12, 1 × 1 μm^2^ areas). Young’s modulus was evaluated using MATLAB programming language from independent tests as the slope of the curve obtained by fitting ∼300 data points in the linear elastic region of the stress–strain curve to a straight line; daily data are shown as mean ± standard (N = 6). Contact angle results are reported as mean ± standard deviation (N = 3 measurements for each substrate). Cell counting was performed on stained nuclei images using MATLAB programming language. For each experimental replica, 2 pictures were taken for each device for every staining condition. Daily data are shown as mean ± standard deviation of independent values extracted from 9 different devices (3 devices for each experimental replica, 2 pictures for each device, N = 18). Each image was first binarized by thresholding, then morphological opening was performed on the binary image using a disk as the structuring element, and finally watershed transforms were applied and areas of connected white pixels were detected (connectivity of 8 pixels). Each area (300 < pixels < 2000) was counted as a single cell. TEER values were measured in triplicate from 3 inserts for each experimental replica; daily data are shown as mean ± standard (2 replicas, N = 18). FITC-dextran concentration values were evaluated from 3 wells for each replica (2 replicas, N = 6). Data were plotted and analyzed by one-way analysis of variance (ANOVA) using MATLAB programming language. Statistical significance was determined when p-value < 0.05.

## 3 Results and Discussion

### 3.1 Combining Roll-to-Roll Gravure Coating and Polymer Phase Separation To Fabricate Ultrathin Porous PDLLA Substrate for Cell Culture

The roll-to-roll gravure coating (Figure 1a) method allows the fabrication of PET–PVA–PDLLA sheets that can be several meters long.^53^ In this process (Figure 1b), a smaller diameter roll, rotating opposite the film, collects the coating solution. A flexible doctor blade removes excess material, ensuring precise transfer onto the film (Figure 1b). Following two consecutive R2R steps and complete solvent evaporation (by dryers in Figure 1a), the PET–PVA–(PDLLA+PS) sheet undergoes polystyrene (PS) etching in cyclohexene (Figure 1c). The PS etching selectively impacts PS without affecting other polymers or the sheet structure: PET is not affected nor corroded, PVA remains undissolved, and PDLLA is unetched (Supporting Information, Figure S4). This is supported by unchanged surface properties of porous PDLLA compared to nonimmersed plain PDLLA nanofilms and quantitative analysis showing PDLLA pore sizes (postetching) not exceeding PS island dimensions (pre-etching), confirming the absence of overetching (Supporting Information, Figure S4). A schematic of the process to fabricate a porous PDLLA nanofilm is summarized in Figure 1c. Thanks to the supporting PET–PVA layer, PDLLA nanofilms can be cut into any desired shape and size. By utilizing a paper tape frame, the rigid PET substrate can be peeled off while maintaining the nanofilm stretched (Supporting Information, Figure S3). As a result, despite its ultrathin thickness, flexibility, and transparency, the film can be easily handled using standard laboratory tweezers (Figure 1c).

**Figure 2:**
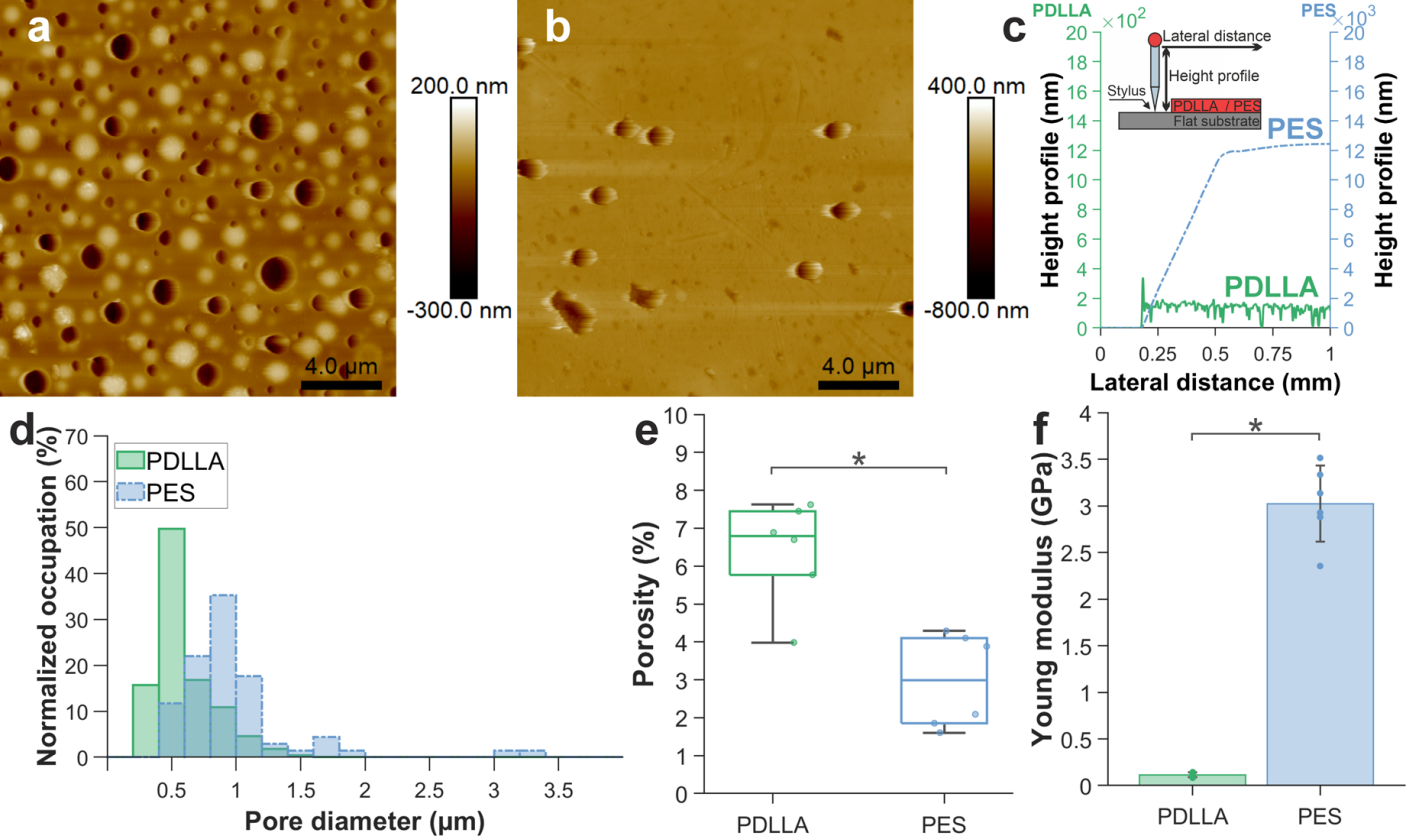
Porous PDLLA nanofilm characterization and comparison with commercially available PES membranes. (a) AFM scan of a 40 mg/mL porous PDLLA nanofilm adherent to a glass coverslip (scale bar: 4 μm). (b) AFM scan of a commercially available PES membrane attached to a glass coverslip. Due to the tip aspect ratio, scans could not reach the coverslip surface across pores (scale bar: 4 μm). (c) Porous PDLLA nanofilm and PES membrane height profile, with inset showing a schematic of thickness measurement. (d) Pore diameter distribution (N = 440 for PDLLA and N = 68 for PES). (e) Pore coverages of porous a PDLLA nanofilm (6 ± 1%) compared to a PES membrane (3 ± 1%) (N = 6 AFM scans, *p < 0.05). (f) Young’s modulus of the PDLLA compared to the PES membrane (bars indicate standard deviation on *N* = 6 measurements, *p < 0.05).

Optical and fluorescence microscopy can be used to assess the integrity of the film, while thickness and porosity can be assessed by AFM scans (Figure 2a–b). The average thickness of the PDLLA nanofilms is 185 ± 22 nm (N = 9) (Figure 2c and Figure S5 in Supporting Information) which is approximately 60 times thinner than the traditional PES membrane (∼11 μm). The submicrometric thickness of the porous PDLLA nanofilms holds significant implications for our coculture model. It promotes favorable conditions for studying paracrine signaling by facilitating a close, almost direct contact between the 2 cell types across the substrate. PDLLA nanofilms also present a homogeneous density of pores in the submicron to nanoscale. PES membrane pores exhibit an average diameter of 0.98 ± 0.49 μm (N = 68), which aligns with the manufacturer’s specifications. More than 50% of the pores have diameters ranging from 0.7 to 1.1 μm. However, the pore size distribution has a second, less prominent peak at higher values. Roughly 2% of the pores have diameters between 3 and 3.4 μm. On the other hand, the porous nanofilms made of PDLLA have smaller average pore diameters of 0.59 ± 0.23 μm (N = 440). More than 80% of the pores fall within the range of 0.2 to 0.8 μm, with none exceeding 1.60 μm in diameter (Figure 2d). This enables their utilization as semipermeable membranes for investigating cell-to-cell signaling without the risk of cell extravasation. The percentage of pore coverage in the porous PDLLA nanofilms (above 3.98%) is also significantly greater (N = 6) than that of PES membranes (3 ± 1%) (Figure 2e). The higher pore density of PDLLA (5 ± 0.9 × 10^7^ cm^–1^, N = 6 AFM scans) compared to PES (3 ± 0.9 × 10^6^ cm^–1^, N = 6 AFM scans) creates more pathways for soluble signaling mediators to travel across the permeable substrate, increasing the diffusion of cell-secreted molecules between the 2 cell types lying on the opposite sides of the substrate.

Basement membrane (BM) porosity is thus not numerically defined in the literature. Characteristic sizes of the cords and filaments that constitute the BM are within 10–150 nm. As an approximation of the pore size, the void space left between those could be approximated to the range of 100s of nm. As an example, the defined value of 92 nm has been determined for human corneal BM with atomic force microscopy but cannot be generalized for different anatomical locations.^59^ While modulations in the topography of the culture substrate are known to influence endothelial cell adhesion and viability, optimal roughness ranges change depending on cell type, conditions and in combination with the other structural properties of the substrate.^60^ Roughness of the porous PDLLA nanofilm is variable (Rq = 12 ± 6 nm and Ra= 10 ± 5 nm, N = 12 1 μm^2^ areas across 3 AFM scans), comparable with PES membranes (Rq = 10 ± 5 nm and Ra= 7 ± 3 nm, N = 12 areas across 3 AFM scans) (Figure S6 in Supporting Information) and aligns with established reference values for cell culture substrates.^61^ Given the impact of substrate mechanics on cell behavior,^62^ an ideal substrate for endothelial cell culture should closely replicate the mechanical properties of the native vascular BM, including Young’s modulus. The Young’s modulus of porous PDLLA nanofilms (0.11 ± 0.03 GPa, N = 6) was 27 times lower than that of commercially available PES membranes (3 ± 0.4 GPa, N = 6) (Figure 2f) and up to 36 times smaller than the Young’s modulus of nonporous PDLLA nanofilms (2–4 GPa) reported in the literature.^63^ This difference between porous and nonporous PDLLA nanofilms is expected as the Young’s modulus of a porous substrate decreases with increasing porosity.^24^ The porous PDLLA nanofilm Young’s modulus is closer to that of the vascular BM,^9,10,64^ which is expected to positively impact cell attachment and growth.^65^ Hydrophilicity significantly influences cellular-material interactions,^66^ and thus the water contact angle was assessed pre and post fibronectin (FN) coating. FN is a glycoprotein in the extracellular matrix (ECM) found to promote growth of endothelial cells.^67^ The decrease in contact angle from 77 ± 4° (N = 3) to 42 ± 4° (N = 3) after coating indicates that FN coating of the porous PDLLA nanofilm increased its hydrophilicity (Figure S7 in Supporting Information), establishing a more suitable condition for cellular adhesion and growth. This behavior remains consistent with the plain PDLLA nanofilm, confirming the unchanged coating process following PS etching (Figure S4 in Supporting Information).

### 3.2 Suspended Porous Polymeric Nanofilms Support Endothelial Cells Growth and Confluent Endothelium Establishment

**Figure 3:**
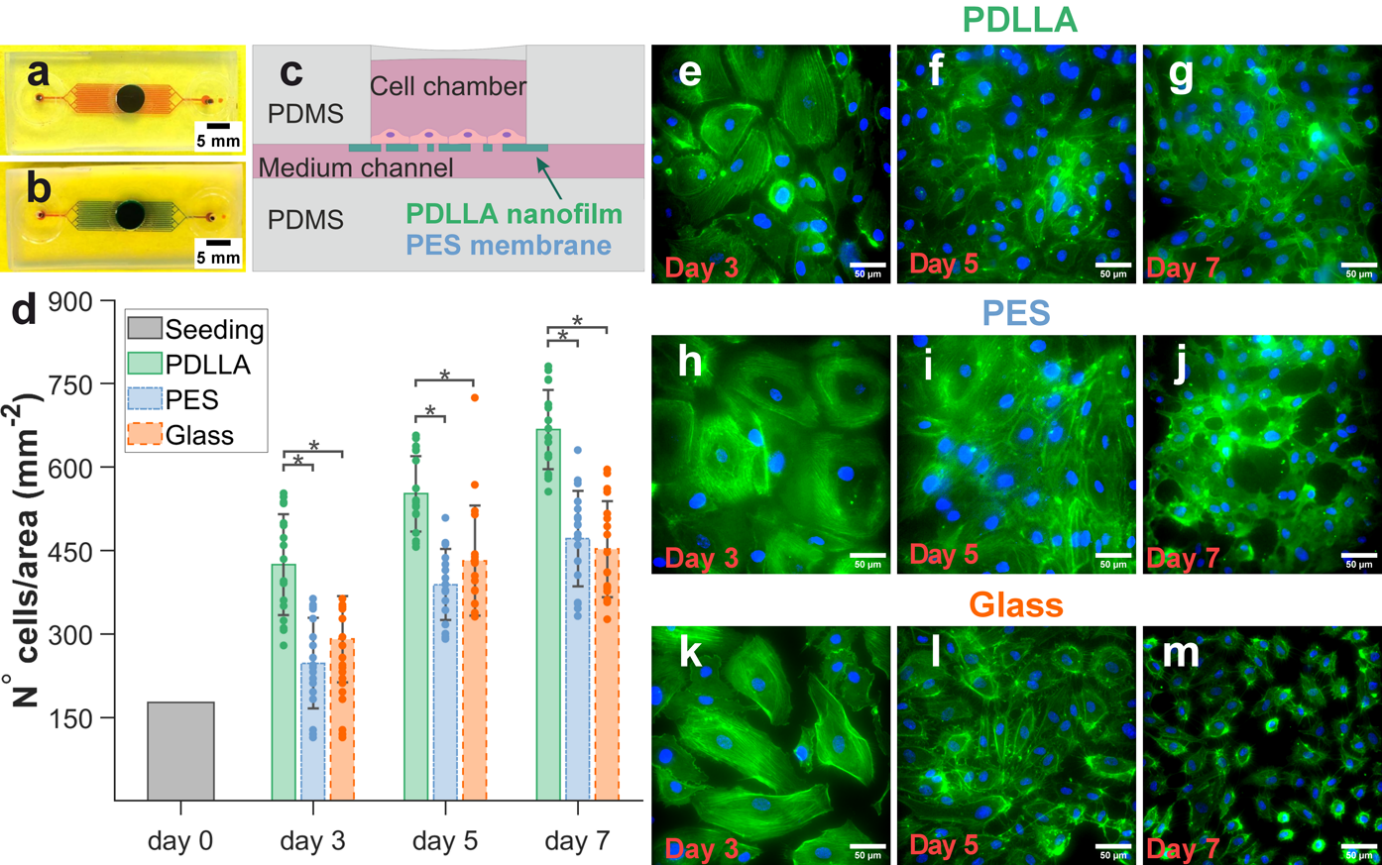
Adhesion, proliferation, and morphology of endothelial cells grown on a porous PDLLA nanofilm embedded in an open microfluidic device. (a–b) Double layer device filled with food coloring; (a) picture taken right after filling the top chamber with blue dye and the bottom chamber with red dye; (b) picture taken 30 min after filling (scale bars: 5 mm). (c) Schematic of the device seeded with HUVECs. (d) HUVECs density on PDLLA nanofilms, PES membrane, and glass bottom device (number of cells/mm^2^). Bars indicate standard deviations on N = 18 counts (3 experimental replicas and 2 images for each device, *p < 0.05). (e–m) HUVECs stained for F-actin (green) and nuclei (blue) on suspended PDLLA nanofilm (e–g), suspended PES membrane (h–j), and glass bottom device (k–m) [(e–m) scale bars: 50 μm].

Previous studies have shown the biocompatibility of ultrathin polymeric films with different cell types, either as plain^68,69^ or porous structures.^46,56^ It is crucial to confirm that the compatibility of porous PDLLA nanofilms persists when they are confined in a microfluidic setup and that they can facilitate the growth of endothelial cells and the formation of a complete endothelial layer, effectively serving as an artificial BM. Combining the natural adhesive properties of PDLLA nanofilms and oxygen plasma activation of surfaces, we established a secure bond between the nanofilm and the PDMS surface. This process allows the assembly of a microfluidic device where the porous film separates two fluidic compartments. Tight sealing and fluidic communication are confirmed, as liquids remain confined in the microfluidic chambers (Figure 3a) and start mixing by diffusion within 10 s after filling (Figure 3b). In the case of the PES membrane, this can be successfully bonded by APTES activation, but the diffusion through the porous structure is slower, with the initial mixing occurring after 2 min and full mixing occurring after 60 min. HUVECs were seeded on the top culturing chamber of an open microfluidic device integrating either PDLLA nanofilms or PES membranes (Figure 3c). This design was used to assess growth and proliferation of cells on the porous membrane and to optimize seeding. Adhesion on the 2 porous substrates is significantly different (N = 18): as shown in Figure 3d, a greater number of viable cells adhere to the porous PDLLA nanofilm within the first 72 h of culture and continue to proliferate with the same trend throughout all 7 days of culture, showing a consistently statistically higher number of cells on the PDLLA film compared to both PES and glass. Over a 7-day period, the HUVECs population on PDLLA porous nanofilms exhibited a proliferation rate of 277 ± 38% across three experimental replicates. In contrast, on PES, the proliferation rate was 166 ± 52%, and on a glass substrate, it was 156 ± 47%. At the seventh day of culture, cell viability is >90% for all of the substrates but significantly higher for porous substrates (97 ± 2% for PDLLA and PES) compared to glass (93 ± 2%) (N = 18) (Figure S8 in Supporting Information).

From day 3 to day 7 of culture, actin filaments (F-actin) are visualized in HUVECs cultured on all substrates (Figure 3 e–m). When cultured on a suspended porous PDLLA nanofilm for 7 days, HUVECs developed the typical cobblestone-like morphology of a mature endothelial monolayer, and as culture progressed, the localization of actin staining shifted from the intracellular body toward the cell periphery, denoting a barrier formation process (Figure 3e–g). This behavior is observed across all substrates during the initial 5 days of culture. However, between day 5 and day 7, there is a slight reversal of this behavior on PES and glass substrates, suggesting a faster dissolution of the endothelial monolayer (Figure 3h–m). On the seventh day of culture, the cytoskeleton of HUVECs grown on PES and glass substrates underwent reorganization, resulting in a smaller and more rounded appearance, indicating the dissolution of the endothelial monolayer (Figure 3j and m).

**Figure 4:**
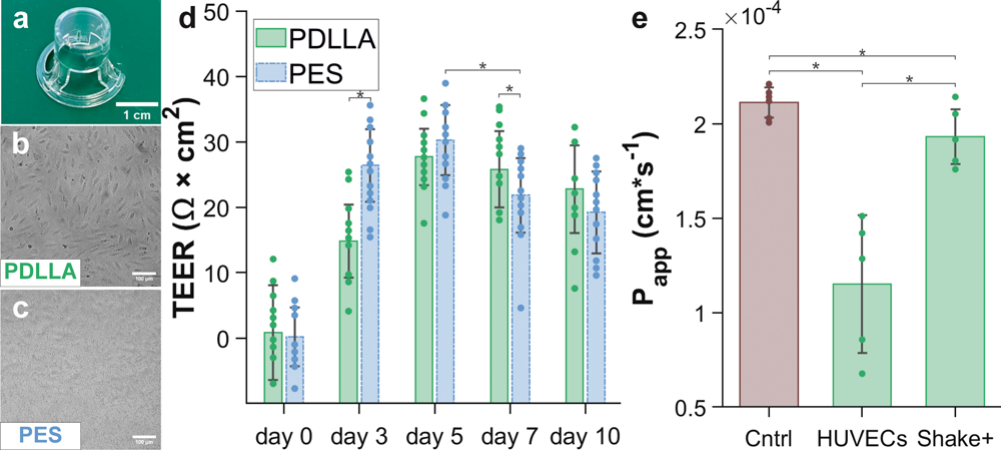
Transendothelial electrical resistance (TEER) measurement of barrier integrity in Transwell inserts mounting porous PDLLA nanofilm or PES membrane: (a) porous PDLLA nanofilm mounted on a Transwell insert (scale bar: 1 cm); (b) HUVECs monolayer formed on the porous PDLLA nanofilm between day 3 and day 5 of culture (scale bar: 100 μm); (c) HUVECs monolayer formed on the porous PES membrane between day 3 and day 5 of culture (scale bar: 100 μm); (d) TEER values (Ohm·cm^2^) for PDLLA porous nanofilm and PES commercially available membrane (bars indicate standard deviation on N = 18 measurements, 2 experimental replicas and 3 measurements for each well, *p < 0.05); (e) results of Transwell permeability assay on PDLLA showed as apparent permeability (*P*_*app*_, cm/s) against FITC-conjugated dextran without cells (Cntrl), at day 5, and after shaking (bars indicate standard deviation on N = 6 Transwell inserts, 2 experimental replicas, 1 measurement for each insert *p < 0.05).

A barrier formation process was observed on PDLLA nanofilms and PES membranes on Transwell inserts and measured by transendothelial electrical resistance (TEER) and permeability assays. Figure 4a shows a porous PDLLA nanofilm successfully mounted on a Transwell insert. The superior transparency of the PDLLA nanofilm in contrast to the PES membrane significantly simplified the process of cell culture and enabled clear observation of cell viability and confluence during TEER measurements (Figure 4b–c). For both substrates, TEER values indicate that the endothelial monolayer is fully formed between the third and fifth day of culture. Although the peak value was achieved more rapidly on PES membranes, the cell barrier function appeared to decline faster on these membranes, while porous PDLLA demonstrated the potential to maintain long-term barrier integrity. Overall, there were no significant differences (N = 18) in the maximum TEER values between the two substrates (Figure 4d). To confirm the formation of an effective cellular barrier, which restricts the passage of large molecules, the Transwell insert culture was replicated to assess the permeability of FITC-conjugated dextran across the endothelial barrier formed on porous PDLLA nanofilms on the fifth day of culture. After 5 days of culture, the permeability decreased by 46%, confirming the establishment of a tight endothelium. Moreover, after shaking the cell monolayer, permeability against FITC-conjugated dextran significantly increases (Figure 4e).

### 3.3 Porous PDLLA Nanofilm Recreates the Basement Membrane in an Endothelial Barrier-on-Chip

**Figure 5:**
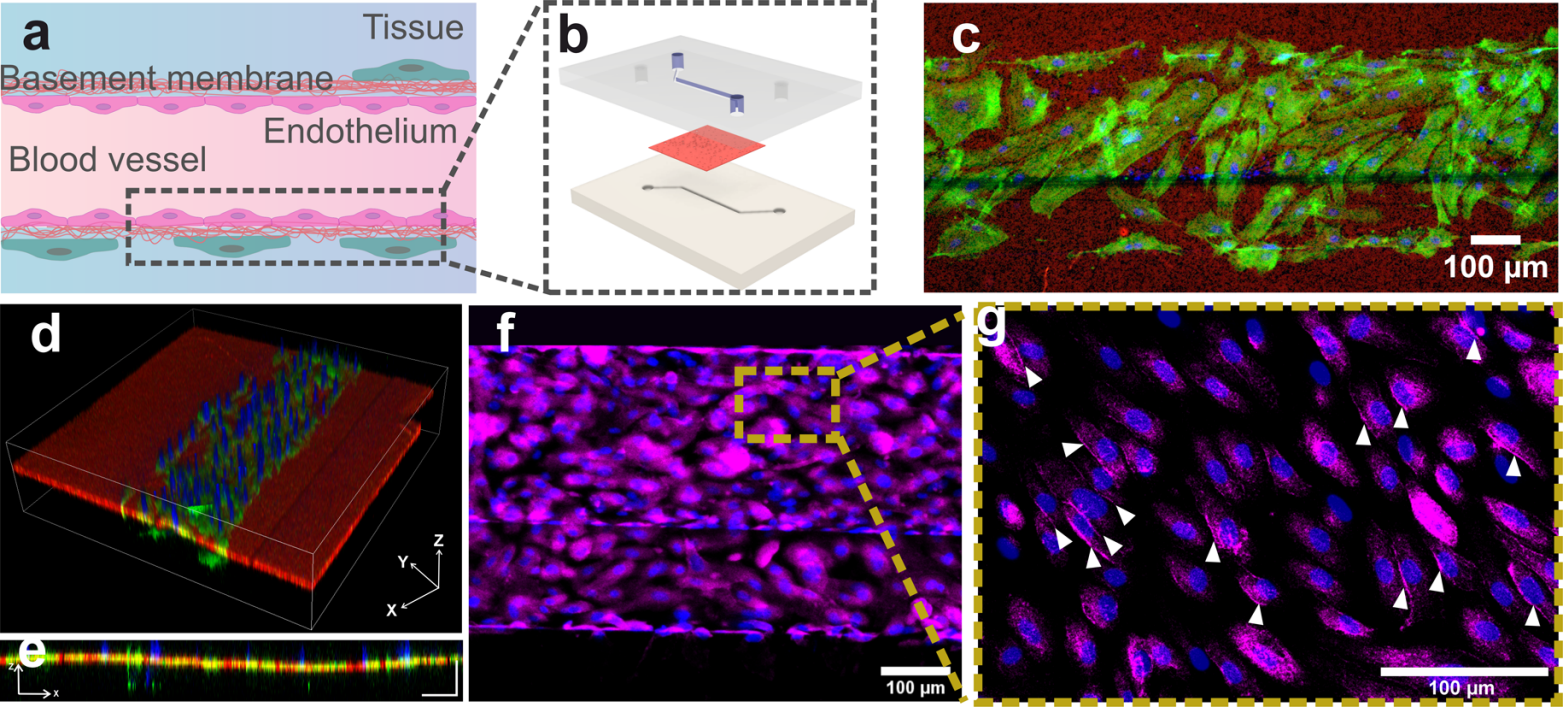
Endothelial barrier-on-chip integrating porous PDLLA nanofilm as basement membrane artificial replica: (a) schematic of endothelial barrier organization; (b) schematic of our endothelial barrier-on-chip with the top microfluidic channel (blue in figure) hosting endothelial cell culture, the PDLLA nanofilm replicating the BM (red in figure), and the bottom microfluidic channel replicating the tissue surrounding the endothelium; (c) HUVECs growing in the device top channel adherent to the fully suspended nanofilm. Nuclei (blue) and F-actin (green) staining of the cells after 5 days of culture on top of the PDLLA nanofilm (red) (scale bar: 100 μm): (d) 3D reconstruction of a confocal z-stack showing the organization of the endothelial barrier-on-chip; (e) lateral view of the reconstructed confocal z-stack of the device (XZ) (scale bars: 100 μm); (f) immunofluorescent staining demonstrating ZO-1 expression (magenta, ZO-1, and blue, nuclei) (scale bar: 100 μm); (g) high magnification image demonstrating ZO-1 peripheral localization (white triangles) (magenta, ZO-1, and blue, nuclei) (scale bar: 100 μm).

In the simplest implementation of an endothelial barrier-on-chip setup, a semipermeable insert is suspended between two adjacent, independently fed microfluidic chambers, namely, the endothelial compartment and the tissue compartment (Figure 5a). Following this approach, a porous PDLLA nanofilm was embedded between two aligned PDMS microchannels (Figure 5b). Following the integration process, no leaks were observed, and the nanofilm appeared flat and fully intact. In this configuration, the insert replicates the structure and functionalities of physiological BMs. When integrated in a double layer microfluidic device, the porous PDLLA nanofilm supports the growth of endothelial cells and physically separates them from the surrounding environment. The integrity of the PDLLA nanofilm was maintained when subjected to a range of flow from 5 to 80 μL/min (videos in Supporting Information). This corresponds to a wall shear stress of 2 Pa (20 dyn/cm^2^), which is higher than the maximum physiological values in microvasculature.^70^ Following exposure to progressively increasing shear stress levels, specifically, 30 min at 1.25 dyn/cm^2^, 30 min at 5 dyn/cm^2^, and 30 min at 20 dyn/cm^2^, the integrity of the device housing the nanofilm was evaluated using bright field and confocal imaging. The results indicated that the film was appropriately suspended and maintained its structural integrity. After the shear stress test, the sealing efficacy of the device was confirmed by the absence of any observable leakages upon filling it with food coloring (Figure S9 in Supporting Information). HUVECs were inoculated in the device, cultured for 5 days under a capillary flow, and checked daily. When the medium is changed (every 12 h), HUVECs experience the maximum shear stress, which amounts to 4 dyn/cm^2^ within the channel.

At the fifth day, cells were fixed and stained for nuclei and F-actin (Figure 5c). Confocal 3D reconstruction of the device showed a porous PDLLA nanofilm correctly suspended between the two channels, free of wrinkles, capable of supporting cell growth while confining it to the upper channel (Figure 5d–e). Zonula occludens-1 (ZO-1) staining and peripheral localization (Figure 5f–g) in higher cell density areas indicate the ongoing development of an endothelial barrier, implying that the channel underlying the nanofilm is prepared to accommodate a second cell type.

**Figure 6:**
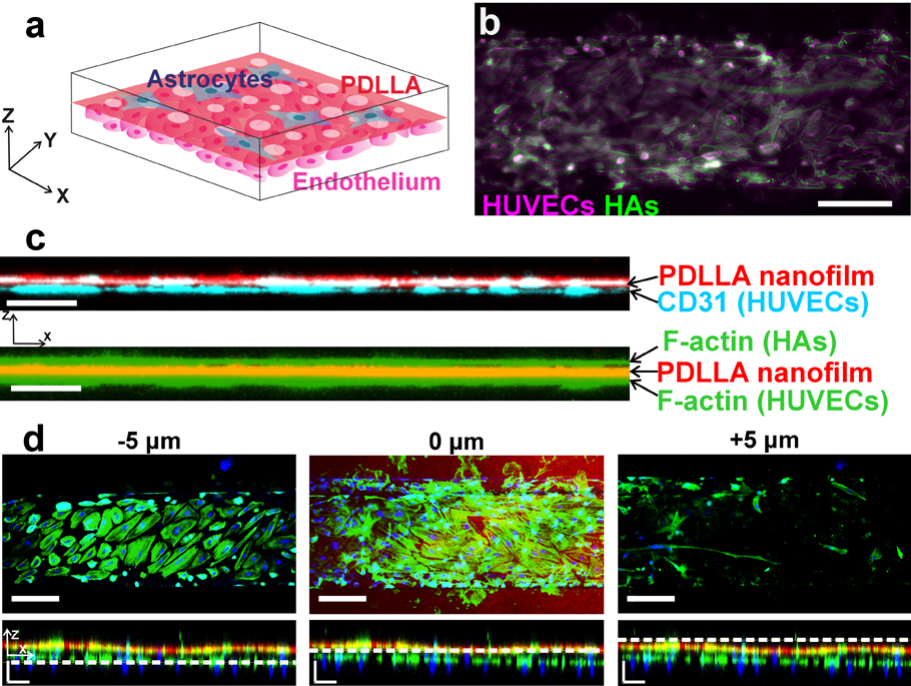
Blood–brain barrier-on-chip prototype integrating porous PDLLA nanofilm: (a) schematic of a portion of the fully assembled BBB-on-chip integrating ultrathin, red-stained porous PDLLA; (b) HUVECs and star-shaped HAs growing in the device, respectively, in the bottom and top channels. Both cell types are adherent to the fully suspended nanofilm. HUVECs (magenta) and HAs (green) are stained for F-actin after 10 and 5 days of culture, respectively (scale bar: 200 μm). (c) Lateral view of the reconstructed confocal z-stack of the device (XZ). Nanofilm highlighted in red (both panel), CD31 in cyan (top panel), and F-actin in green (bottom panel) (scale bars: 50 μm). (d) Fluorescent images of HUVECs and HAs arranged layer by layer along the Z-axis with the 0 μm position corresponding to the red-stained nanofilm location. Bottom panels: XZ lateral views, dashed white lines mark the position along the Z-axis (scale bars: 100 μm).

To establish a coculture in the device, we introduced human astrocytes (HAs) into the upper channel once a mature endothelial layer had developed over 5 days, as depicted in Figure 6a. Endothelial cells and astrocytes are key cellular components of the functional unit of the blood–brain barrier (BBB). After an additional 5 days of culture, cells were fixed and stained for nuclei, F-actin (Figure 6b), and CD31, which is a widely used marker for HUVECs and not expressed in astrocytes. A 3D confocal reconstruction of the device reveals the nanofilm effectively isolating the two cell types (Figure 6c), with CD31 (shown in light blue) exclusively localized beneath the nanofilm in the endothelial compartment. As shown in Figure 6d, the extremely thin thickness of the film results in the close proximity of the two cell types, with flat astrocytes nearly fused with the membrane pores. Observation of distinct cell morphologies on opposite sides of the nanofilms revealed polygonal and rounded HUVECs beneath the PDLLA nanofilms and star-shaped HAs adhering to the other side (Figure 6d). The 2:1 ratio of endothelial cells vs astrocytes loaded in the device allows limiting the overgrowth of astrocytes in the device, mimics the real characteristics of the BBB, and results in a limited number of astrocytes stretching on a confluent layer of endothelial cells.

## 4 Conclusion

This study presented ultrathin, flexible, and transparent porous PDLLA nanofilms as viable substitutes for synthetic basement membranes (BMs) in endothelial barrier models. When integrated as a physical separation between different cell types, porous PDLLA nanofilms facilitate close and direct cell-to-cell communication without the risk of cell extravasation. This unique environment for cell-to-cell interaction is given by their submicrometric thickness, homogeneous distribution of nanoscale pores, and higher pore density compared to commercially available polyester membranes. Their mechanical properties more closely resemble the native vascular BM, with a significantly lower Young’s modulus. This leads to enhanced cell attachment and growth and to the formation of a tight endothelium in short-term culture. Furthermore, the integration of PDLLA nanofilms within a double layer microfluidic device demonstrates their ability to sustain integrity under fluidic pressure, making them suitable for modeling the dynamic vascular microenvironment *in vitro* in healthy or pathological conditions. The formation of the endothelium, already showing tight-junction formation under quasi-static conditions, could be further reinforced by a continuous perfusion to show polarization and to control proliferation. Additionally, the coculture of a second cell type in close proximity with the endothelial cells would also support the endothelial barrier functions. As a characteristic example, the coculture with astrocytes represents a first step toward the development of a more specific blood–brain barrier for modeling and studying neurodegenerative diseases and for testing pharmaceutical treatments. The nanometric thickness and porosity of the film clearly resemble the real BM. These are essential to reduce the impact of 3D gels, thick scaffolds, and porous micrometric membranes often used in blood–brain barrier (BBB) models, which introduce artifacts not always taken into consideration, influence the diffusion of chemicals and the migration of cells, and reduce the communication between cells on the two sides of the barrier.

This initial successful implementation of the model will now be followed by the optimization of the manufacturing process to increase the yield and throughput of device fabrication. Since the roll-to-roll gravure coating process allows us to produce ultrathin films with limited restrictions in terms of area, and multiple microfluidic channels can be fabricated on a single silicon wafer, future work will be dedicated to the parallel assembly of multiple devices using a single interdigitated film. This step will be essential to facilitate the use of this the new organ-on-chip design for critical applications such as BBB-on-chip, opening new possibilities for drug screening, disease modeling, and personalized medicine.

## Data Availability

The data underlying this study are openly available in University of Leeds at 10.5518/1473, reference number 1473.

## Supporting Information Available

Step-by-step schematic of the process to mount a PDLLA nanofilm on a Transwell insert; standard curve of dextran concentration and fluorescence; step-by-step schematic of the fabrication process to assemble a double layer microfluidic PDMS device integrating a porous PDLLA nanofilm; effects of cyclohexene treatment on polymeric sheet composed of PET, PVA and PDLLA; height profiles of porous PDLLA nanofilms; surface roughness comparison between porous PDLLA nanofilms and commercially available PES membranes; contact angle measurements of uncoated and fibronectin (FN) coated porous PDLLA nanofilm; LIVE/DEAD assay results for HUVECs growing on porous PDLLA nanofilms and PES commercially available membranes stretched across microchannels and glass substrates; effect of shear stress exposure on porous PDLLA nanofilm suspended between 2 aligned microfluidic channels (PDF)
Videos of a PDMS double channel microfluidic device integrating porous PDLLA nanofilm exposed to different levels of shear stress: 1.25 dyn/cm^2^ (5 μL/min), 5 dyn/cm^2^ (20 μL/min), and 20 dyn/cm^2^ (80 μL/min); slowed down video of a PDMS double channel microfluidic device integrating PDLLA porous nanofilm exposed to 20 dyn/cm^2^ shear stress (video speed: 0.1× recorded speed) (ZIP)

## Abbreviations

OoC: organ-on-chip
ECM: extracellular matrix
BM: basement membrane
PES: polyester
PC: polycarbonate
PDMS: polydimethylsiloxane
BBB: blood–brain barrier
R2R: roll-to-roll
PDLLA: poly(d,l-lactic acid)
HUVEC: human umbilical vein endothelial cell
PS: polystyrene
PET: poly(ethylene terephthalate)
PVA: poly(vinyl alcohol)
AFM: atomic force microscope
FN: fibronectin
HBSS: Hank’s balanced salt solution
TEER: transendothelial electrical resistance
FITC: fluorescein isothiocyanate
APTES: (3-aminopropylsilane)triethoxysilane
PBS: phosphate-buffered saline
HA: human astrocyte
FBS: fetal bovine serum
AM: astrocytes medium
F-actin: actin filaments
ZO: zonula occludens
CD31: platelet endothelial cell adhesion molecule
