# Supplementary material for: Porous Polymeric Nanofilms for Recreating the Basement Membrane in an Endothelial Barrier-on-Chip
Source: ACS Appl Mater Interfaces. 2024 Feb 28;16(10):13006–17. doi: 10.1021/acsami.3c16134 (PMC10941076; doi:10.1021/acsami.3c16134)
Supplement: Supplementary file 1 — am3c16134_si_001.pdf [file am3c16134_si_001.pdf]

Supporting Information

Porous polymeric nanofilms for recreating the basement membrane in an endothelial barrier-on-chip

Elena Mancinelli[12], Nanami Zushi[3], Megumi Takuma[3], Chalmers Chi Cheng Chau[124], George Parpas[125], Toshinori Fujie[36], Virginia Pensabene[12]*

[1] School of Electronic and Electrical Engineering and Pollard Institute, University of Leeds, Leeds, LS2 9JT UK

[2] Bragg Centre for Materials Research, University of Leeds, Leeds, LS2 9JT, UK

[3] School of Life Science and Technology, Tokyo Institute of Technology, B-50, Nagatsuta-cho, Midori-ku, Yokohama, 226-8501, Japan

[4] School of Molecular and Cellular Biology and Astbury Centre for Structural Molecular Biology, University of Leeds, LeedsLS2 9JT, UK

[5] St James University Hospital, Welcome Trust Brenner Building, University of Leeds, LS9 7TF, UK

[6] Living Systems Materialogy (LiSM) Research Group, International Research Frontiers Initiative (IRFI), Tokyo Institute of Technology, R3-23, 4259 Nagatsuta-cho, Midori-ku, Yokohama, 226-8503, Japan

*Corresponding author: Virginia Pensabene, V.Pensabene@leeds.ac.uk

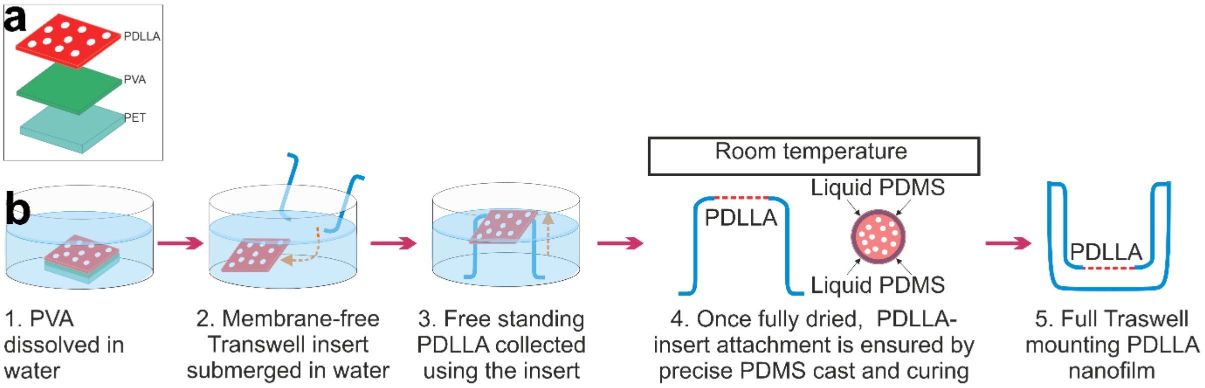

Figure S1. Step-by-step process to mount a PDLLA porous nanofilm on a Transwell insert: a) schematic of the polymeric sheet prior to the mounting process. The sheet is formed by a supporting layer in PET, a sacrificial layer in PVA, and the PDLLA nanofilm; b) schematic illustration of the mounting process. 1. The sheet (shown in panel a) is immerged in water for 10 minutes to completely dissolve the PVA layer; 2. the free-standing nanofilm is then scooped with a membrane-free insert and 3. removed from the water; 4. the assembly (Transwell mounting the nanofilm) is then allowed to dry at room temperature overnight inside a biological cabinet and secured in place using liquid PDMS to firmly fix the edges to the plastic insert. 5. After 24 hours the nanofilm is ready to use for coating and cell seeding.

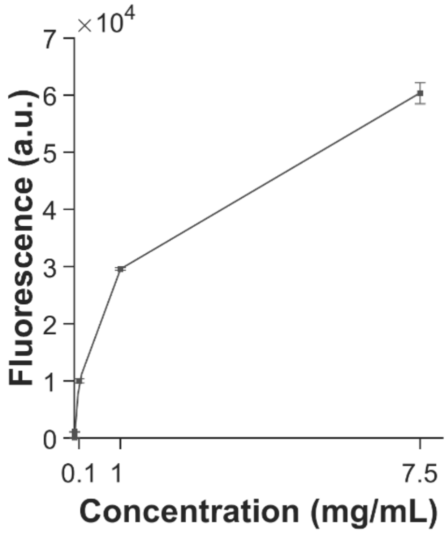

Figure S2. Standard curve for dextran (for each point, N=3).

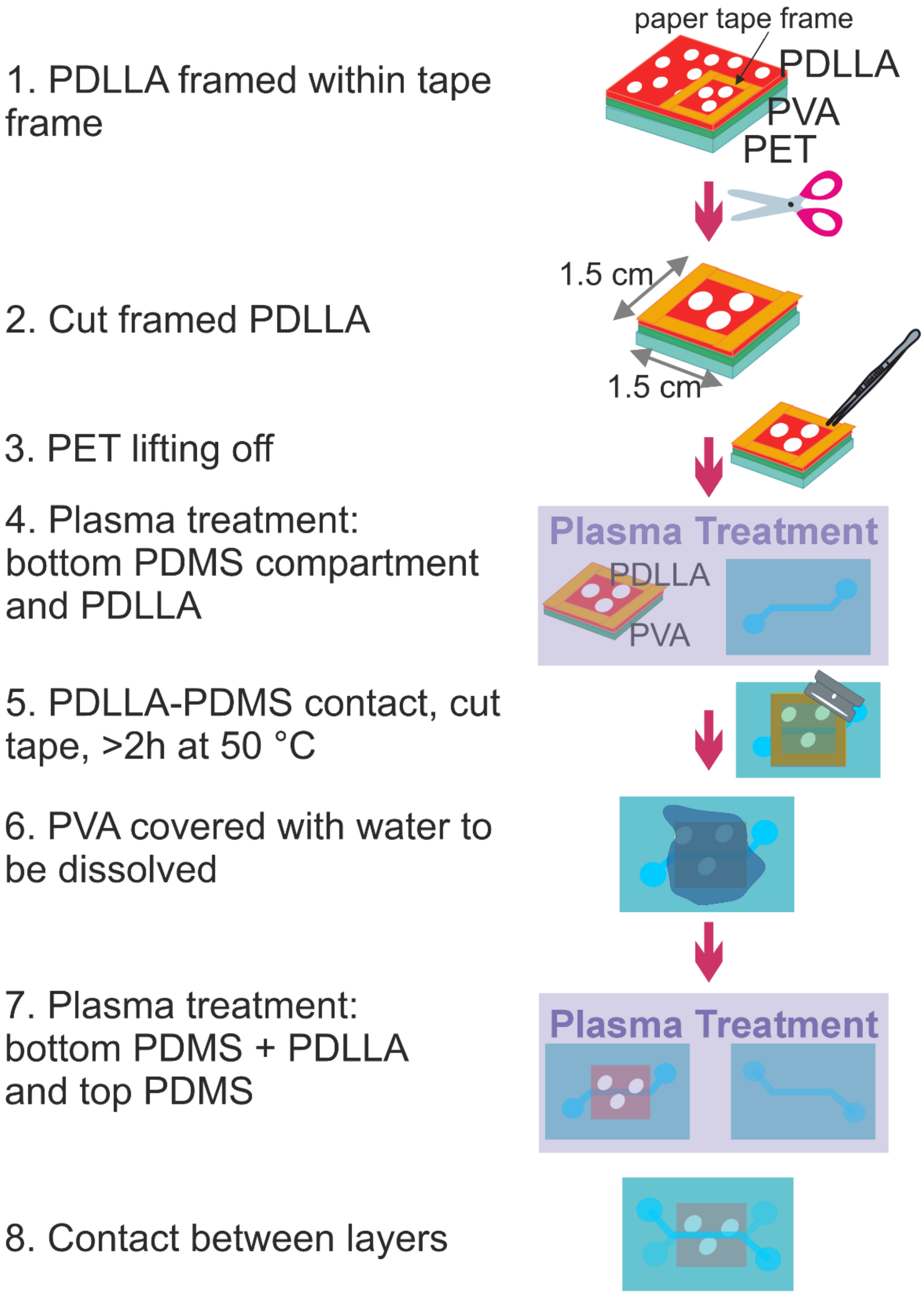

Figure S3. Step-by-step fabrication process of a double layer microfluidic PDMS device integrating a porous PDLLA nanofilm: 1. PDLLA is framed within a paper tape frame and 2. cut accordingly; 3. the tape frame serves to lift off the PVA-PDLLA sheet form the PET supporting layer; 4. PDMS bottom layer and PDLLA nanofilm undergo oxygen plasma treatment; 5. PDLLA is brought into contact with PDMS and the 2 layers are left at 50°C for 2 hours; 6. the PVA is dissolved and the PDMS-PDLLA assembly is left at room temperature for 24 hours until fully dried (not shown); 7. top PDMS layer and PDMS-PDLLA assembly undergo oxygen plasma treatment before being brought into contact to compose the full device. The full device is then left for at least 2 hours at 50°C

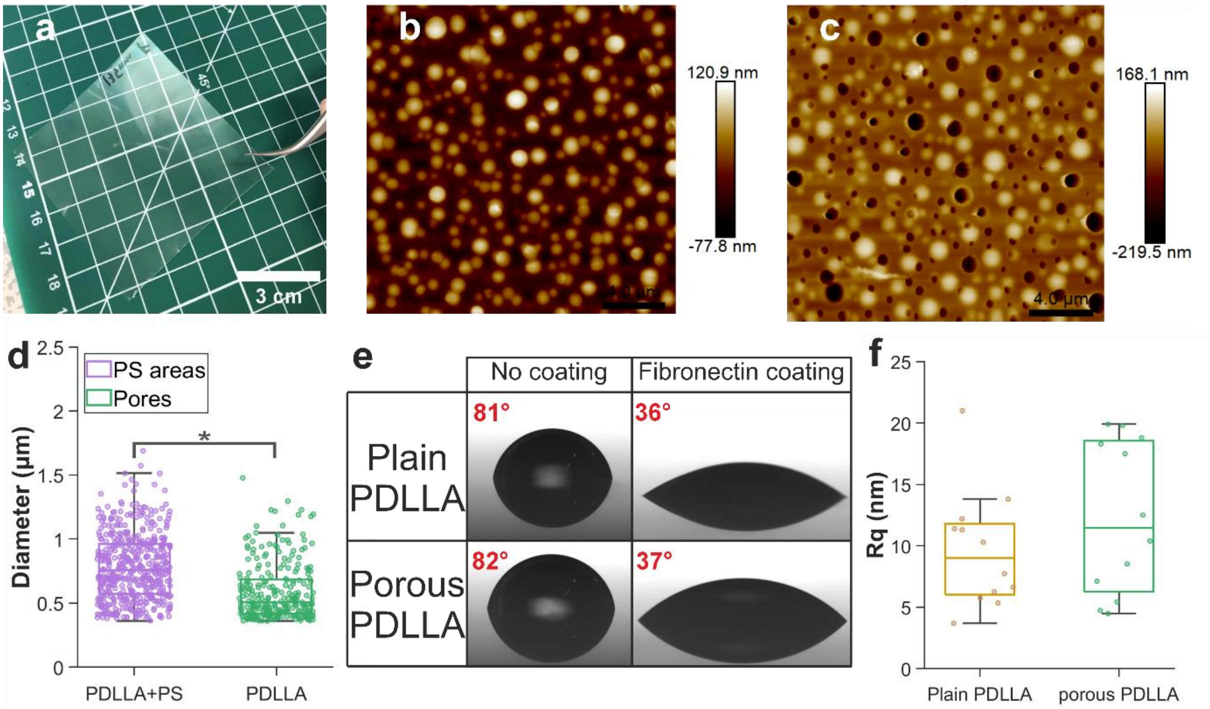

Figure S4. Effects of cyclohexene treatment: a) Polymeric sheet handled with tweezers, after treatment with cyclohexane to dissolve the PS. b) AFM images of the PDLLA layer before and c) after treatment with cyclohexane (scale bar 4 µm). (d) Diameter of the PS islands (pre-etching) and the pores in the PDLLA nanofilm (post- etching) (Ø PS islands: 0.8 ± 0.3 µm, across 3 AFM scans, Ø pores:0.6 ± 0.2 µm, across 3 AFM scans). e) Representative images of contact angle measurements on plain PDLLA nanofilms before and after coating with fibronectin (top row) and of porous PDLLA nanofilms before and after coating (second row); f) Roughness of plain (Rq =10 ± 5 nm) and porous PDLLA nanofilm (Rq =12 ± 6 nm) (for both Rq values N=12 1×1 µm^2 areas across 3 AFM scans).

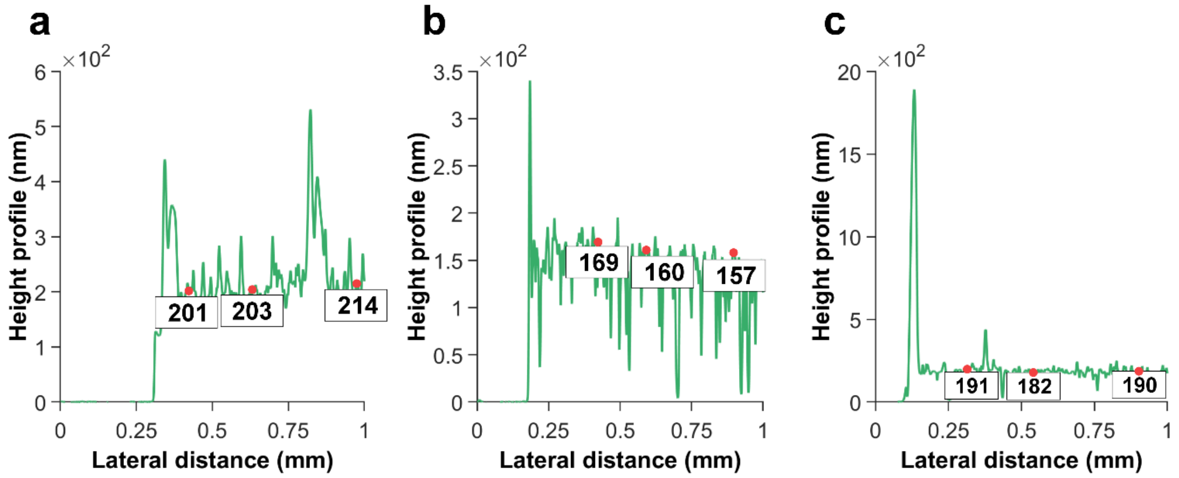

Figure S5. Porous PDLLA nanofilm height profiles: a-b) height profiles of PDLLA nanofilms fabricated from 40mg/mL solution of PDLLA and polystyrene in ethyl acetate; c) heigh profile of a PDLLA nanofilm fabricated from 40 mg/mL of PDLLA and polystyrene and 10^{-4} mg/mL of Nile Red in ethyl acetate. Points used to estimate average height of the nanofilms are highlighted in each panel (a,b,c).

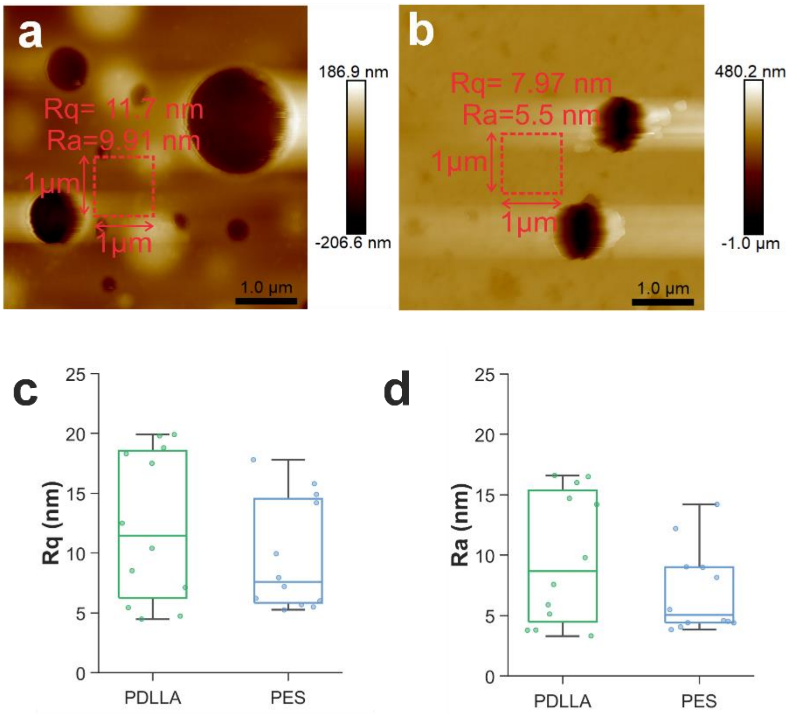

Figure S6. Surface roughness comparison between porous PDLLA nanofilm and commercially available PES membrane: a) AFM height scan of PDLLA porous nanofilm (scale bar: 1 μm); b) AFM height scan of PES membrane (scale bar: 1 μm). The delineated red squares (a,b) are illustrative examples of the region of interest used to calculate the surface roughness between pores. c-d) corresponding roughness of PDLLA nanofilm (Rq = 12 ± 6 nm, Ra= 10 ± 5 nm, N=12), and PES membrane (Rq =10 ± 5 nm and Ra= 7 ± 3 nm) (for all measurements, N=12 1×1 μm^2 areas across 3 AFM scans).

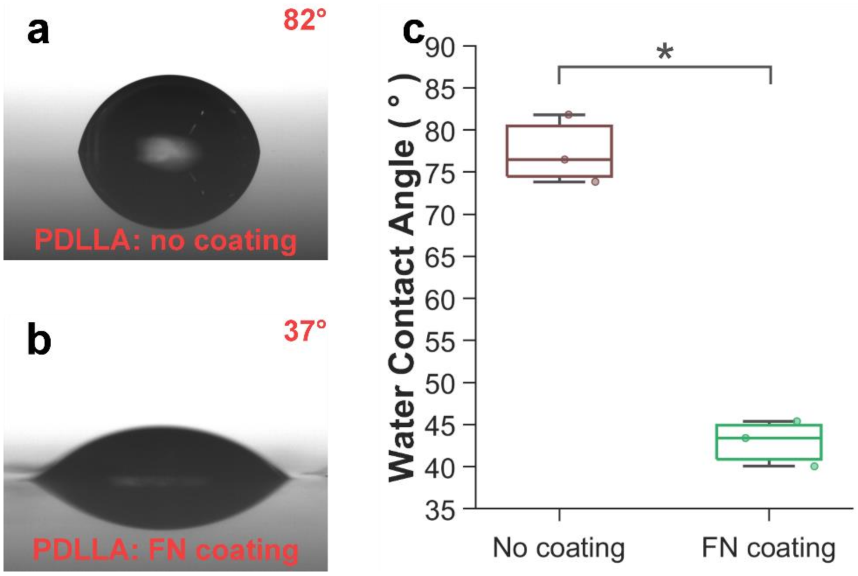

Figure S7. Contact angle measurements of uncoated and fibronectin (FN) coated porous PDLLA nanofilm: a) uncoated porous PDLLA nanofilms exhibit a higher hydrophobicity compared to b) FN-coated porous PDLLA nanofilms; c) contact angle measurement results

showing an angle of 77 ± 4° (N=3) for uncoated porous PDLLA nanofilm and of 42 ± 4° (N=3) for FN-coated porous PDLLA nanofilm.

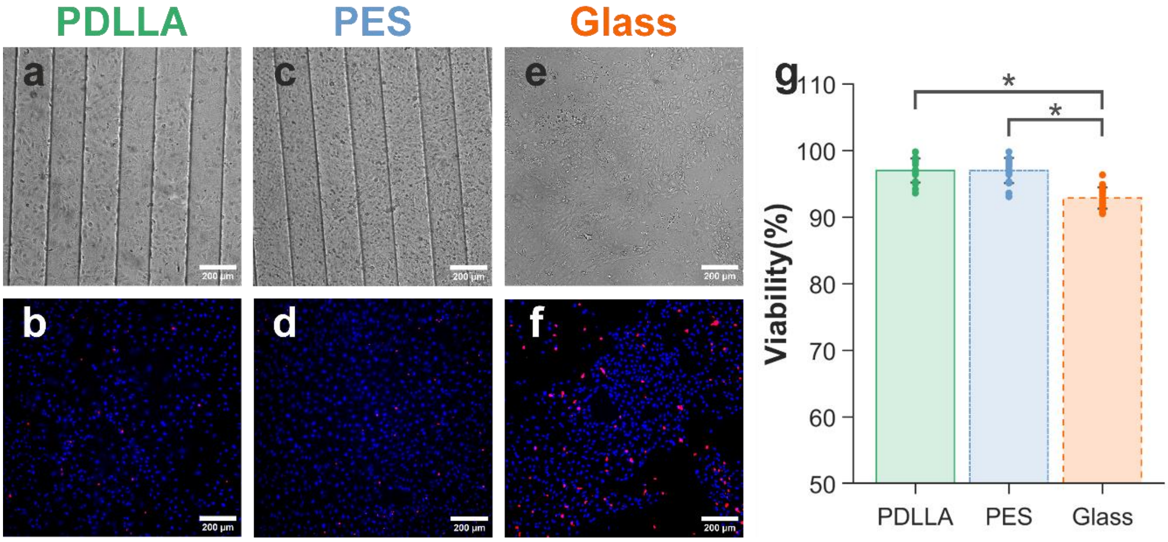

Figure S8. LIVE/DEAD assay for HUVECs growing on porous PDLLA nanofilm, PES membrane stretched across microchannels and glass substrate: a) HUVECs after 7 days of culture on suspended porous PDLLA nanofilm (scale bar: 200 µm) and b) relative LIVE/DEAD assay (red for dead cells and blue for live cell nuclei, scale bar: 200 µm); c) HUVECs after 7 days of culture on suspended PES membrane (scale bar: 200 µm) and d) relative LIVE/DEAD assay (red for dead cells and blue for live cell nuclei, scale bar: 200 µm); e) HUVECs after 7 days of culture on glass substrate (scale bar: 200 µm) and f) relative LIVE/DEAD assay (red for dead cells and blue for live cell nuclei, scale bar: 200 µm); g) cell viability results on the 3 substrates express as percentage of live cells (97± 2 % for PDLLA and PES and 93± 2% compared to glass) (N=18 sets of images across 3 replicas of the experiment with 3 devices of each type per replica).

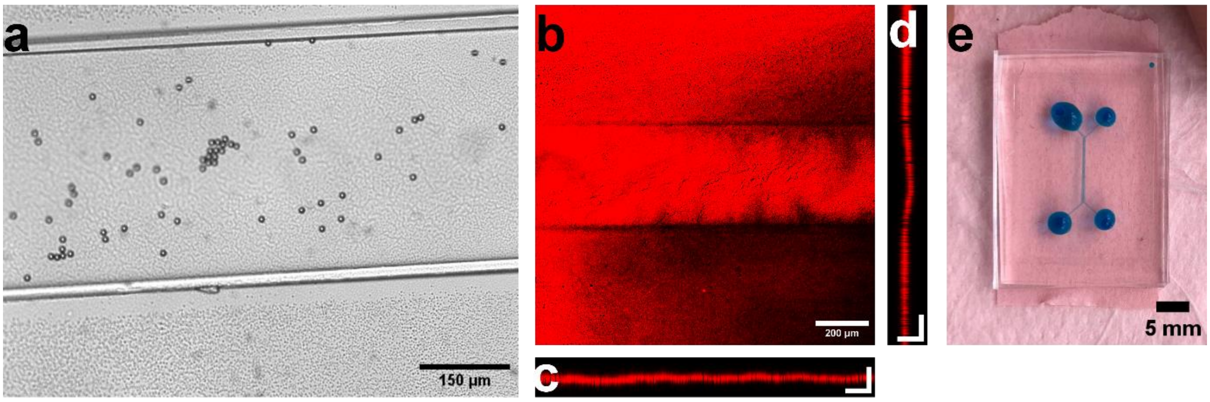

Figure S9. Effect of shear stress exposure on porous PDLLA nanofilm suspended between 2 aligned microfluidic channels**:** a) bright field image depicting intact PDLLA nanofilm after shear stress exposure through flow of a polystyrene bead solution (scale bar: 150 µm); b-c-d) confocal reconstruction of the suspended pours PDLLA nanofilm shear stress exposure. The red stained PDLLA porous nanofilm appears correctly suspended and intact from top (XY, b) (scale bar: 200 µm) and lateral views (XZ in c, YZ in d) (scale bars: 100 µm); e) device integrating porous PDLLA nanofilm showing no leaks after loading with blue food colour and flow application. Highest imposed shear stress: 20 dyn/cm^2.

Corresponding Author

Virginia Pensabene – School of Electronic and Electrical Engineering and Pollard Institute; Bragg Centre for Materials Research, University of Leeds, Leeds, LS2 9JT, U.K.;

ORCID: 0000-0002-3352-8202; Email: V.Pensabene@leeds.ac.uk.
